# Protective Effects of *Luffa cylindrica* Leaf Infusion Against Cisplatin and CCl_4_‐Induced Hepatorenal Toxicity in Rats: Biochemical, Histopathological, and Molecular Docking Analysis

**DOI:** 10.1155/jt/8845909

**Published:** 2026-04-06

**Authors:** Takoua Ben Hlel, Anouar Feriani, Nadia Khelifi, Zouhaier Abbes, Issam Smaali

**Affiliations:** ^1^ Laboratory of Protein Engineering and Bioactive Molecules (LIP-MB 11ES24), INSAT-BP, Carthage University, Centre Urbain Nord Cedex, Tunis, 1080, 676, Tunisia, carthage.edu; ^2^ Higher Institute of Biotechnology of Beja (ISBB), University of Jendouba, Beja, 9000, Tunisia, uj.rnu.tn; ^3^ Laboratory of Biotechnology and Biomonitoring of the Environment and Oasis Ecosystems, Faculty of Sciences of Gafsa, University of Gafsa, Gafsa, 2112, Tunisia, ugaf.rnu.tn; ^4^ Field Crops Laboratory, National Institute for Agricultural Research of Tunisia (INRAT), Carthage University, Hedi Karray Street El Menzah, 1004, Ariana, Tunisia, carthage.edu

**Keywords:** carbon tetrachloride, cisplatin, *Luffa cylindrica*, hepatotoxicity, molecular docking, nephrotoxicity, oxidative stress, phenolic compounds, Wistar rats

## Abstract

This study demonstrated the antioxidant potential of *Luffa cylindrica* leaf infusion (LLI) in alleviating oxidative damage induced by cisplatin and carbon tetrachloride (CCl_4_) in Wistar rats. Phytochemical analysis confirmed the presence of bioactive compounds, such as protocatechuic acid (PCA; 5.83 mg/g DW), naringenin (5.54 mg/g DW), chlorogenic acid (CA; 4.90 mg/g DW), and ascorbic acid (4.87 mg/g DW). In vivo results showed that LLI administration by gavage significantly improved kidney and liver functions, particularly at a dose of 40 mg/kg, as evidenced by serum biochemical markers for both organs. Furthermore, LLI pretreatment restored superoxide dismutase (SOD) and CAT activity while reducing MDA levels, indicating a protective effect against oxidative stress. Histopathological analysis revealed that LLI treatment reduced structural damage in liver and kidney tissues, confirming its protective capacity. Molecular docking studies revealed that naringenin, PCA, and CA exhibit strong binding affinities to SOD, potentially enhancing its activity synergistically. These findings suggest that LLI, rich in natural antioxidants, may serve as a promising therapeutic candidate for mitigating oxidative stress–induced organ damage, warranting further investigation in clinical settings.

## 1. Introduction

Oxidative stress is a critical factor in the development of numerous pathological conditions, including kidney and liver damage, neurodegenerative diseases, and cancer. The excessive production of reactive oxygen species (ROS) disrupts cellular homeostasis, leading to oxidative damage to lipids, proteins, and DNA [1]. This oxidative damage is a key contributor to the progression of these diseases. Cisplatin and carbon tetrachloride (CCl_4_) are commonly used toxic agents to induce toxicity, as they are known to cause significant oxidative stress and organ damage, particularly in the liver and kidneys [2, 3]. The damage caused by these agents also mimics the oxidative stress and organ damage associated with many chemotherapeutic drugs and harmful substances, highlighting the need for effective therapeutic strategies to mitigate such effects. Superoxide dismutase (SOD) and catalase (CAT) are key enzymes that protect cells from oxidative damage. SOD in particular is the first line of defense in the enzymatic antioxidant system. It catalyzes the conversion of superoxide radicals into hydrogen peroxide and oxygen [4, 5]. Increased SOD activity enhances cellular resilience to oxidative stress, making it a promising target for therapies aimed at reducing oxidative damage. The potential of natural antioxidants to strengthen the body’s endogenous defense mechanisms against oxidative stress has drawn a lot of attention. These compounds, often found in medicinal plants, possess strong antioxidant and free radical scavenging abilities [6]. By neutralizing reactive oxygen species (ROS), they shield cells from oxidative damage and support overall cellular health. In addition to their role in alleviating liver diseases, such as chronic hepatitis C, alcoholic hepatitis, and non‐alcoholic fatty liver disease (NAFLD), antioxidants have also shown promise in mitigating kidney damage [7, 8]. Studies have demonstrated that antioxidants can help prevent or alleviate conditions like chronic kidney disease (CKD), acute kidney injury (AKI), and nephrotoxicity induced by drugs such as cisplatin by reducing oxidative stress and preserving kidney function [7, 9].

*Luffa cylindrica* is a versatile plant widely recognized for its fibrous fruit, commonly used as a natural sponge. However, the leaves of *L. cylindrica*, a valuable but underutilized resource, are rich in bioactive compounds like phenolics and flavonoids with strong antioxidant properties. *L. cylindrica* is a domesticated species, regularly cultivated in several Asian and African countries. The valorization of its aerial parts represents a sustainable and renewable biomass source [10, 11].

Methanolic extracts of the leaves have been found to contain several important phenolic compounds, including gentisic acid, ferulic acid, protocatechuic acid (PCA), *p*‐coumaric acid, quercetin, and apigenin‐7‐glucoside. The leaves are edible and can be consumed steamed, lightly fried, or prepared as an herbal tea [12, 13].

This study aims to evaluate the protective effects of *L. cylindrica* leaf infusion (LLI) against oxidative stress‐induced organ damage in a Wistar rat model. The study combines in vivo assessments of antioxidant enzyme activity with in silico molecular docking of the major phenolic compounds in LLI: naringenin, chlorogenic acid (CA), and PCA with SOD. By integrating biochemical analyses and molecular docking simulations, this research seeks to provide a deeper understanding of how *L. cylindrica* leaf infusion protects against oxidative stress‐induced toxicity.

## 2. Material and Methods

### 2.1. Plant Material and Infusion Preparation

*L. cylindrica leaves* were harvested from the Kairouan region in Tunisia (36°44′N, 09°11′E) in May 2024. The plant material was identified by Pr. Chokri Messaoud, and a voucher specimen (VC Ind. 48/24) was deposited at the herbarium of the National Institute of Applied Sciences and Technology of Tunis (INSAT, Tunis). Subsequently, they were thoroughly washed and allowed to air‐dry in the shade at ambient temperature (25 ± 2°C) in a well‐ventilated area for a period of 15 days. The plant material was finely ground into a powder using an electric grinder. Then, 1 g of the powdered sample was infused with 50 mL of boiling distilled water. The obtained infusion was filtered and freeze‐dried, then stored at room temperature until further use.

### 2.2. Phenolic Compounds Analysis by HPLC–DAD

Phenolic compound characterization of LLI was conducted using high‐performance liquid chromatography (HPLC) with an Agilent Technologies 1260 system (Germany), equipped with a reversed‐phase C18 analytical column of 4.6 × 100 mm and 3.5‐μm particle size (Zorbax Eclipse XDB C18). DAD detection was performed in the range of 200–400 nm. The column temperature was maintained at 25°C throughout the analysis. The flow rate was set to 0.4 mL/min, and the injection volume was 2 μL. The mobile phases consisted of Milli‐Q water with 0.1% formic acid (mobile phase B) and methanol (mobile phase A). Chromatographic conditions were optimized as follows: 0–5 min: 10% A‐90% B; 5–10 min: 20% A‐80% B; 10–30 min: 30% A‐70% B; 30–40 min: 50% A‐50% B; 40–45 min: 60% A‐40% B; 45–50 min: 70% A‐30% B; 50–55 min: 90% A‐10% B; 55–60 min: 50% A‐50% B; and at 60 min: 10% A‐90% B. The phenolics identification was performed by comparing their retention time and the UV spectra with those of pure standards. The characteristics of the calibration curves obtained by injecting known concentrations of several standard compounds were used to calculate the limits of detection and for quantitative analysis.

### 2.3. In Vivo Assays

#### 2.3.1. Animals

Mature male Wistar rats (7–8 weeks old, 230–250 g) were procured from SIPHAT (Tunisia). Male Wistar rats were used to reduce biological variability linked to female hormonal fluctuations that can affect metabolism and oxidative stress, as this model is well established for reproducible responses [14]. The rats were kept in standard cages at the Faculty of Sciences in Gafsa, Tunisia, under controlled conditions of temperature (23°C), relative humidity (55%), and a 12‐h light/dark cycle for a week before treatment. During acclimation, the animals were provided with free access to clean water and were fed with standard food pellets (SNA‐Sfax, Tunisia). The study was approved by the Research Ethics Committee of the University of Gafsa, Tunisia (UG/FSG_01/25), and all experimental procedures were conducted in accordance with established ethical guidelines and the ARRIVE (Animal Research: Reporting of In Vivo Experiments) guidelines.

#### 2.3.2. Dose Selection and Preliminary Safety Screening

A preliminary safety study was performed to assess the safety profile and determine the effective dosing range of LLI extract. Thirty rats were randomly divided into six experimental groups (*n* = 5 per group). Five groups received oral doses of LLI dissolved in physiological saline at 5, 10, 15, 20, and 40 mg/kg/day for 14 consecutive days, while a control group received saline alone.

Animals were individually observed during the first 4 h post‐administration and subsequently monitored daily for clinical signs of toxicity, behavioral alterations, mortality, and changes in body weight. No adverse effects or mortality were observed at the highest tested doses (20 and 40 mg/kg); therefore, these were selected as the therapeutic doses for the subsequent in vivo experiments.

#### 2.3.3. Experimental Design

A total of 54 Wistar rats were randomly allocated into nine distinct groups, each comprising six animals [15]. The groups underwent various treatments as detailed as follows:

− **Control group:** received corn oil.
− **LLI1 treated group:** received LLI dissolved in corn oil at a dosage of 20 mg/kg body weight (LLI1).
− **LLI2 treated group:** received LLI dissolved in corn oil at a dosage of 40 mg/kg body weight (LLI2).
− **Cisplatin treated group:** received cisplatin at a dosage of 13 mg/kg body weight.
− **CCL_4_ treated group:** received CCl_4_ at a dosage of 1 mL/kg body weight.
− **LLI1 pretreatment + toxin groups:** received LLI1 pretreatment, followed by cisplatin or CCl_4_ administration.
− **LLI2 pretreatment + toxin group:** received LLI2 pretreatment, followed by cisplatin or CCl_4_ administration.

The LLI doses were administered to the animals 7 days prior to the initiation of cisplatin or CCL4 treatment and continued daily throughout the study. Following the 7‐day pretreatment period, cisplatin was administered intraperitoneally, while CCl_4_ was administered by oral gavage, twice weekly for 4 weeks to induce toxicity. Twenty‐four hours after the final toxin administration, animals were anesthetized by intraperitoneal injection of pentobarbital (50 mg/kg) and subsequently sacrificed by decapitation.

#### 2.3.4. Blood Collection and Tissue Preservation

Blood samples were collected via cardiac puncture, placed into a tube containing a heparin anticoagulant, and centrifuged at 3000 rpm for 10 min. The resulting serum was stored at −20°C. Kidney and liver tissues were promptly extracted, rinsed in an ice‐cold saline solution, and divided into two parts. One part was fixed in a 4% buffered paraformaldehyde phosphate solution overnight at 4°C and subsequently embedded in paraffin. The remaining tissue was stored at −80°C [9].

#### 2.3.5. Serum Biochemical Markers

Creatinine (CR), blood urea nitrogen (BUN), and urea levels were measured to assess kidney function. Aspartate aminotransferase (AST), gamma‐glutamyl transferase (GGT), alanine aminotransferase (ALT), lactate dehydrogenase (LDH), alkaline phosphatase (ALP), total cholesterol (TC), and triglycerides (TG) levels were quantified to evaluate liver function. All biomarker levels were determined using commercial reagent kits (Biomaghreb, Tunisia) according to the manufacturer’s instructions.

#### 2.3.6. Oxidative Stress Biomarkers

##### 2.3.6.1. Tissue Homogenate

One gram of each hepatic and renal tissue sample was homogenized using an Ultra‐Turrax homogenizer (IKA Ultra‐Turrax T25 basic homogenizer, Germany) in a solution containing 2 mL of ice‐cold lysis buffer (Tris‐buffered saline, pH 7.4) for 15 min. Each sample was then centrifuged (9000 × g at 4 °C for 30 min). The supernatants obtained were stored at −80°C until use.

##### 2.3.6.2. Oxidative Status Assessment

CAT activity was determined according to Aebi (1984) [16]. The reaction mixture (1 mL) had 100 mM phosphate buffer (pH 7), 100 mM H_2_O_2,_ and 20 μL of kidney or liver extracts. H_2_O_2_ decomposition was assessed at 25°C by measuring the decrease in absorbance at 240 nm for 1 min. Enzyme activity was expressed in μmol H_2_O_2_ decomposed/min/mg protein. The SOD activity was evaluated using the method described by Marklund and Marklund [17], and results were expressed as units (U)/mg of protein. Lipid peroxidation assessment in both organs followed the method outlined by Draper and Hadley [18]. In brief, 100 μL of each kidney and liver homogenate was combined with 100 μL of trichloroacetic acid (TCA, 5%). After centrifugation at 4000 × g for 10 min, 100 μL of the resulting supernatant was mixed with 200 μL of thiobarbituric acid (TBA) reagent (0.67%) and then incubated for 15 min on a boiling water bath. The quantification of lipid peroxidation levels was determined as thiobarbituric acid reactive substances (TBARS), expressed as malondialdehyde (MDA), and estimated in nmol/mg of tissue.

#### 2.3.7. Histopathological Analysis

The paraffin‐embedded fixed hepatic tissues were cut into 4–6‐μm‐thick pieces with a Leica RM2115 microtome (Leica, Bensheim, Germany) for various histopathological colorations. Hematoxylin–eosin (H&E) staining was used to elucidate the morphological structure. Different images of the liver or kidney from each experimental group were captured under a Bresser Erudit Basic Bino microscope (Bresser, Brignoles, France), with a magnification range of 40X to 400X, to assess histological changes [19].

#### 2.3.8. Molecular Docking

A molecular docking study was conducted to explore the binding modes of three major polyphenols from LLI: naringenin, CA, and PCA with SOD. PyRx software (Virtual Screening Tool, Version 0.8), developed by Trott and Olson, was used for this study. PyRx integrates several tools, including AutoDock Vina, AutoDock 4.2, Mayavi, Open Babel, and Python‐based utilities. The three‐dimensional (3D) structure of SOD (PDB ID: 1CB4) was obtained from the RCSB Protein Data Bank (https://www.rcsb.org/) and converted to PDBQT format for docking. The 3D structures of the polyphenols were downloaded from PubChem (https://pubchem.ncbi.nlm.nih.gov/). ChimeraX (Version 1.8), developed by the UCSF Resource for Biocomputing, Visualization, and Informatics, was employed to prepare the docking input files. This involved assigning atomic charges, merging non‐polar hydrogens, and defining rotatable bonds for the ligands. Gasteiger charges were applied, and the macromolecule was similarly prepared for docking. The docking grid box was centered on the enzyme’s active site at x: 15.003, y: 69.747, and z: 15.311, with dimensions of 39.34 × 63.05 × 25.0 Å, while the exhaustiveness parameter was set to 8. The docking poses were scored using the AutoDock Vina scoring function, and the best‐scoring conformation was selected for further analysis. The binding interactions between the polyphenols and SOD were visualized using PyMOL 3.0.4 and further analyzed with ChimeraX.

### 2.4. Statistical Analysis

Statistical analysis was performed using Statistica 12 for Windows. Experimental results were expressed as mean ± SEM for in vivo results (*n* = 6) and as mean ± SD (*n* = 3) for HPLC–DAD results. The normality of data distribution was verified using the Shapiro–Wilk test. Statistical significance was determined using one‐way ANOVA followed by Tukey’s post hoc test, where *p*‐values < 0.05 were considered statistically significant.

## 3. Results

### 3.1. Phenolic Compounds Profile

**FIGURE 1:**
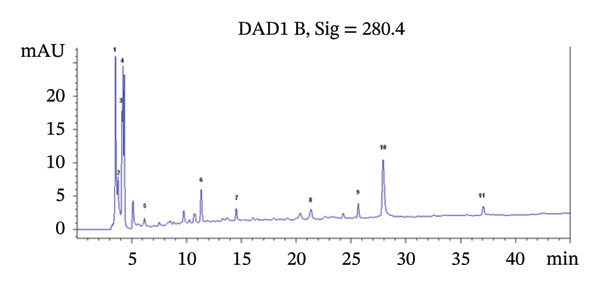
HPLC–DAD profile of bioactive compounds identified in *Luffa cylindrica* leaf infusion: (1) ascorbic acid, (2) gentisic acid, (3) 4‐hydroxybenzoic acid, (4) protocatechuic acid, (5) gallic acid, (6) chlorogenic acid, (7) caffeic acid, (8) ferulic acid, (9) quercetin dihydrate, (10) naringenin, (11) kaempferol.

HPLC–DAD analysis revealed the presence of 11 compounds in *L. cylindrica* dry leaf infusion, including 7 phenolic acids, 3 flavonoids, and ascorbic acid (Figure 1). PCA (5.83 mg/g DW), naringenin (5.54 mg/g DW), CA (4.90 mg/g DW), and 4‐hydroxybenzoic acid (3.01 mg/g DW) were the most abundant compounds (Table 1).

**TABLE 1:** Phenolic compounds and ascorbic acid contents of LLI detected by HPLC–DAD.

Peak number	Retention time (min)	Compound name	Content in mg/g DW
1	3.525	Ascorbic acid	4.87 ± 0.01
2	3.754	Gentisic acid	2.43 ± 0.02
3	4.124	4‐Hydroxybenzoic acid	3.01 ± 0.02
4	4.210	Protocatechuic acid	5.83 ± 0.03
5	6.176	Gallic acid	0.43 ± 0.02
6	11.341	Chlorogenic acid	4.90 ± 0.01
7	14.5419	Cafeic acid	0.28 ± 0.02
8	21.353	Ferulic acid	1.63 ± 0.03
9	25.654	Quercetine dihydrate	0.87 ± 0.03
10	27.930	Naringenin	5.54 ± 0.02
11	37.069	Kaempherol	0.68 ± 0.03

*Note:* Content values are expressed in mean ± SD (n = 3).

Abbreviation: RT, retention time.

### 3.2. Kidney Function Biomarkers

The effects of different treatments on the kidney function of rats were evaluated, as indicated by the levels of BUN, urea, and CR described in Table 2. The control group, which received no treatment, exhibited baseline levels of these indicators. Rats administered LLI at doses of 20 mg/kg of body weight (LLI1) and 40 mg/kg of body weight (LLI2) generally exhibited biochemical profiles comparable to the control group, confirming the absence of nephrotoxicity. However, a significant decrease (*p* < 0.05) in BUN levels was observed in the LLI2 group compared to the control, suggesting a potential improvement in baseline renal filtration. In contrast, rats treated with cisplatin, a known nephrotoxin, showed significantly (*p* < 0.05) elevated levels of BUN, urea, and CR, indicating impaired kidney function. Interestingly, the pre‐treatment with LLI followed by the co‐treatment with cisplatin and LLI (at 20 mg/kg of body weight and particularly at 40 mg/kg of body weight) resulted in BUN, urea, and CR levels significantly lower than those in the cisplatin group, suggesting a dose‐dependent improvement in kidney function (Table 2).

**TABLE 2:** Effects of *Luffa cylindrica* leaf infusion (LLI) on renal function markers in cisplatin‐induced toxicity in Wistar rats.

	**BUN**	**Urea**	**Creatinine**

Control	7.23 ± 1^b^	52.02 ± 2^a^	64.78 ± 2^a^
LLI1	7.16 ± 1^b^	51.79 ± 2^a^	64.13 ± 1^a^
LLI2	6.17 ± 0.5^a^	50.68 ± 1^a^	63.06 ± 1^a^
CPL^∗^	19.63 ± 0.4^e^	127.16 ± 3^d^	105.82 ± 4^c^
CPL + LLI1	14.99 ± 1^d^	104.27 ± 2^c^	86.77 ± 2^b^
CPL + LLI2	11.80 ± 1^c^	82.71 ± 2^b^	65.03 ± 3^a^

*Note:* ^∗^CPL: Cisplatin. Values were expressed as the mean ± SEM (*n* = 6). Different letters (a, b, c, d, e) indicate statistically significant differences (*p* < 0.05) between groups (*p* < 0.05. Tukey test for multiple comparisons).

### 3.3. Liver Function Biomarkers

The levels of ALT, AST, ALP, GGT, LDH, TC, and TG were determined in rats with different treatments (Table 3). Assessment of hepatic markers confirmed the safety of the infusion. While AST, LDH, TC, and TG levels in LLI‐treated groups remained statistically comparable to the control, rats receiving LLI2 exhibited a significant reduction (*p* < 0.05) in ALT, ALP, and GGT, suggesting a potential improvement in basal liver function. The administration of CCl_4_ caused liver toxicity as expected, which was shown by the significant increase (*p* < 0.05) in all liver biomarkers compared to the control. Noticeably, LLI pre‐ and co‐treatment mitigated some of the CCl4‐induced liver damage, with the effect being more pronounced at the higher LLI dose, as evidenced by a statistically significant decrease (*p* < 0.05) in all biochemical markers compared to the CCl4‐only group.

**TABLE 3:** Effects of *Luffa cylindrica* leaf infusion (LLI) on hepatic function markers in CCl_4_‐induced toxicity in Wistar rats.

	**ALT**	**AST**	**ALP**	**GGT**	**LDH**	**TC**	**TG**

Control	43.07 ± 1^b^	84.94 ± 2^a^	34.12 ± 1^b^	31.23 ± 1^b^	191.82 ± 3^a^	61.01 ± 1^a^	62.82 ± 1^a^
LLI1	42.95 ± 2^b^	83.19 ± 2^a^	33.59 ± 1^b^	30.96 ± 2^b^	194.61 ± 2^a^	60.17 ± 2^a^	62.20 ± 2^a^
LLI2	35.42 ± 1^a^	82.78 ± 1^a^	27.63 ± 1^a^	27.43 ± 1^a^	190.37 ± 2^a^	59.81 ± 2^a^	61.15 ± 0.5^a^
CCl_4_	135.38 ± 1^e^	245.53 ± 3^d^	88.53 ± 2^d^	88.29 ± 2^d^	636.75 ± 4^d^	125.86 ± 3^d^	133.52 ± 2^d^
CCl_4_ + LLI1	78.52 ± 1^d^	170.14 ± 2^c^	49.37 ± 1^c^	49.79 ± 1^c^	398.35 ± 3^c^	82.69 ± 2^c^	102.22 ± 2^c^
CCl_4_ + LLI2	63.39 ± 0.8^c^	153.57 ± 3^b^	33.56 ± 1^b^	31.21 ± 1^b^	372.43 ± 3^b^	70.07 ± 2^b^	90.54 ± 3^b^

*Note:* Values were expressed as the mean ± SEM (*n* = 6). Different letters (a, b, c, d, e) indicate statistically significant differences (*p* < 0.05) between groups (*p* < 0.05, Tukey test for multiple comparisons).

### 3.4. Lipid Peroxidation and Oxidative Enzymes Status

The impact of various treatments on SOD and CAT levels in the liver and kidneys of rats was evaluated (Table 4). Administration of LLI at both doses resulted in oxidative marker levels comparable to the control group, with no significant differences observed (*p* < 0.05). However, rats treated with cisplatin or CCL_4_ alone had significantly lower (*p* < 0.05) enzymatic activity levels of SOD and CAT compared to the control. In contrast, the pretreatment with LLI1 and LLI2 significantly (*p* < 0.05) restored most of the activity of both enzymes in both organs in a dose‐dependent manner. MDA levels were also examined in the kidney and liver. A nearly twofold increase was detected in the liver of rats treated with CCl_4_, while a more than threefold increase was observed in the kidneys of rats given cisplatin compared to the control and LLI groups (*p* < 0.05). When pretreated with LLI, whether at a dose of 20 mg/kg or 40 mg/kg body weight, a significant reduction in MDA levels was observed in both organs compared to the toxin‐only groups (*p* < 0.05).

**TABLE 4:** Effects of *Luffa cylindrica* leaf infusion (LLI) on oxidative stress and antioxidant enzyme activities in liver and kidney following toxin‐induced damage.

	**Liver**			**Kidney**		
	**MDA**	**SOD**	**CAT**	**MDA**	**SOD**	**CAT**

Control	43.57 ± 2^a^	8.50 ± 0.4^d^	63.32 ± 2^c^	24.19 ± 1^a^	6.19 ± 1^c^	12.01 ± 2^c^
LLI1	40.96 ± 1^a^	8.14 ± 1^d^	63.80 ± 2^c^	23.53 ± 2^a^	5.97 ± 1^c^	12.01 ± 2^c^
LLI2	41.39 ± 2^a^	8.94 ± 1^d^	62.24 ± 3^c^	25.96 ± 1^a^	6.11 ± 0.7^c^	12.41 ± 1^c^
Toxin^∗^	71.1 ± 3^b^	1.72 ± 0.2^a^	39.32 ± 2^a^	76.54 ± 1^c^	1.50 ± 0.5^a^	4.82 ± 1^a^
Toxin^∗^ + LLI1	41.61 ± 2^a^	6.37 ± 0.5^b^	45.24 ± 2^b^	52.06 ± 1^b^	2.52 ± 0.8^b^	6.66 ± 3^b^
Toxin^∗^ + LLI2	43.54 ± 1^a^	7.49 ± 1^c^	58.75 ± 1^c^	45.19 ± 1^b^	2.46 ± 0.2^b^	9.19 ± 2^c^

*Note:* Values were expressed as the mean ± SEM (*n* = 6). Different letters (a, b, c, d, e) indicate statistically significant differences (*p* < 0.05) between groups (*p* < 0.05. Tukey test for multiple comparisons).

^∗^Toxin: CCl_4_ or cisplatin for hepatic or renal toxicity damage, respectively.

### 3.5. Histopathological Examination

**FIGURE 2:**
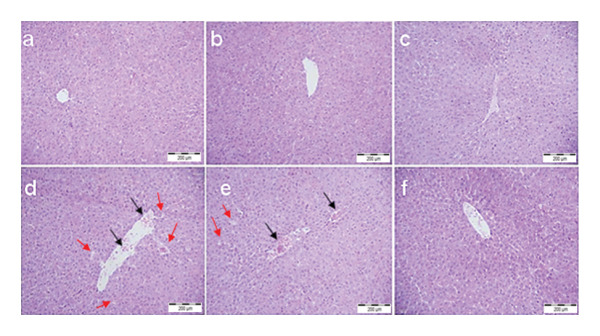
The histopathological examination of liver sections stained with hematoxylin–eosin (G x 200) for the different groups: a: Control, b: LLI1, c: LLI2, d: CCL4, e: CCL4 + LLI1, and f: CCL4 + LLI2. Black arrows: congestion of the centrilobular vein and infiltration of inflammatory cells. Red arrows indicated dilated sinusoids.

**FIGURE 3:**
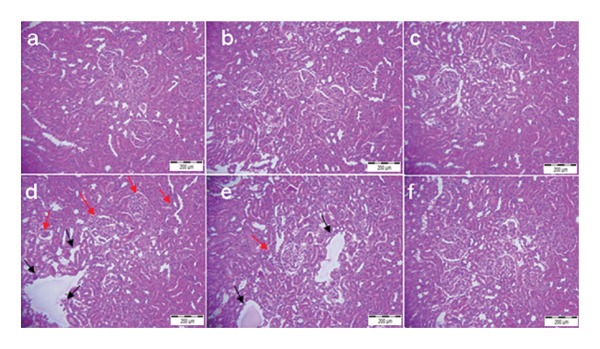
The histopathological examination of kidney sections stained with hematoxylin–eosin (G x 200) for the different groups: a: Control, b: LLI1, c: LLI2, d: CPL, e: CPL + LLI1, and f: CPL + LLI2. Black arrows: dilation and severe epithelial lesions. Red arrows: glomerular and tubular inflammation.

Figures 2 and 3 illustrate the histological examination results (H&E staining) of the control and treated groups. The hepatic and renal tissues of both the control group and the groups treated with LLI1 and LLI2 exhibited normal structures. In the liver (Figure 2), the sections revealed polyhedral hepatocytes arranged around the central vein, with normal sinusoids in between. In contrast, rats intoxicated with CCl_4_ displayed notable abnormalities, including dilated sinusoids, significant congestion of the centrilobular vein, and infiltration of inflammatory cells. However, pre‐treatment with LLI at both doses markedly alleviated the liver injuries induced by CCl_4_. Histopathological analysis showed that the glomerulus and tubular regions of the kidneys appeared normal in the control and untreated rats. In contrast, rats treated with cisplatin exhibited glomerular and tubular inflammation, accompanied by dilation and severe epithelial lesions (Figure 3). However, rats treated with LLI extract displayed minimal renal necrotic lesions and moderate structural alterations in the proximal and distal tubular epithelia, with few to no signs of inflammation.

### 3.6. Molecular Docking

**FIGURE 4:**
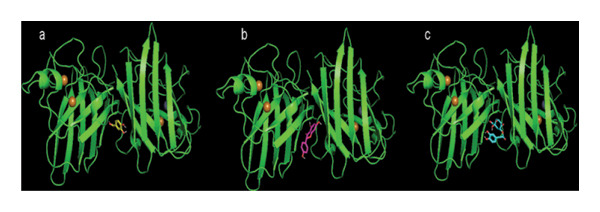
3‐D docking complexes of superoxide dismutase with ligands: (a) protocatechuic acid, (b) naringenin, and (c) chlorogenic acid.

The docking study was conducted to investigate the potential interactions between SOD and major bioactive compounds from LLI, such as naringenin, PCA, and CA (Figure 4). By analyzing hydrogen bonds, hydrophobic contacts, and binding affinities, this study aims to provide insights into how these compounds may stabilize SOD and contribute to the enzyme’s enhanced activity observed in vivo under CCl_4_ and cisplatin‐induced toxicity. The molecular docking analysis revealed favorable binding affinities of the phenolic compounds against SOD. CA exhibited the most potent binding energy of −8.1 kcal/mol, indicating a strong likelihood of effective interaction with SOD. Naringenin followed closely with a binding energy of −7.7 kcal/mol, while PCA displayed the least favorable binding energy of −6.0 kcal/mol (Table 5). Chlorogenic acid forms hydrogen bonds with CYS 6, ASN 51, and LYS 9, while the hydrophobic interactions are predominantly focused around residues valine (VAL 7, VAL 146). Naringenin establishes a hydrogen bond with VAL 7 and several hydrophobic interactions, while PCA interacts through hydrogen bonds with VAL 7 and VAL 146.

**TABLE 5:** Binding energies, hydrogen bonds, and hydrophobic interactions between SOD and major phenolic compounds in LLI.

Target	Ligand	Binding energy (kcal/mol)	Hydrogen contacts	Hydrophobic contact
SOD	Chlorogenic acid	−8.1	CYS 6. ASN 51. LYS 9	VAL 146 H ↔ C VAL 146 CB ↔ C VAL 7 H ↔ C VAL 7 N ↔ O VAL 146 CG2 ↔ C VAL 7 CG2 ↔ C VAL 7 CB ↔ C
	Naringenin	−7.7	VAL 7	VAL 146 CB ↔ C VAL 146 H ↔ C VAL 146 CG2 ↔ C VAL 7 H ↔ C LYS 9 CB ↔ C GLY 145 CA ↔ C ASN 51 CB ↔ C GLY 10 N ↔ C
	Protocatechuic acid	−6.0	VAL 7. VAL 146	VAL 146 CB ↔ C VAL 146 CG2 ↔ C

## 4. Discussion

Nephrotoxicity and hepatotoxicity induced by agents like cisplatin and CCl_4_ pose significant health risks, primarily mediated through oxidative stress and inflammation [9, 20]. While natural biomolecules have gained attention for mitigating these effects [21, 22], this study explored LLI as a natural source of bioactive compounds to address nephrotoxicity and hepatotoxicity. The results highlight LLI’s therapeutic potential, which is attributed to its rich composition of phenolic compounds such as CA, naringenin, PCA, caffeic acid, and gallic acid and its high ascorbic acid content (4.87 mg/g DW). In a previous study conducted on *L. cylindrica* leaves’ methanolic extract from samples collected from Mahdia and Medenine in Tunisia [12], the phenolic profiles varied significantly, highlighting the influence of environmental and genetic factors. In the current study, in addition to previously reported phenolic compounds, caffeic acid and gallic acid were detected in the leaf infusion from the Kairouan region (Tunisia). The HPLC analysis also revealed significant amounts of ascorbic acid (4.87 mg/g DW), indicating a high vitamin C content, which is substantially higher than the level (0.069 mg/g DW) previously reported by Saliu et al. [22].

In the context of nephrotoxicity, pre‐treatment with LLI (particularly at 40 mg/kg) offered a significant protective effect on kidney function. The observed reduction in BUN, urea, and CR levels suggests that LLI ameliorates cisplatin‐induced renal damage. A similar nephroprotective effect was observed by Abigail and Metuaghan, who reported a significant decrease in CR and urea in diabetic rats treated with *L. cylindrica* [23]. While cisplatin induces AKI by initiating excessive inflammation and programmed cell death [3], many polyphenols are proven to modulate these pathways. Among these bioactive polyphenols, naringenin and PCA are abundant in LLI. In fact, naringenin was found to suppress the intestinal edema in acute colitis induced in mice, while PCA was able to inhibit the adhesion of monocytes to mouse aortic endothelial cells and also reduce NF‐κB activity [24, 25].

Building upon the observed improvements in kidney function, the effects of LLI on liver biomarkers also demonstrate its potential as a protective agent. Treatment with LLI, at both doses, successfully mitigated some of the liver damage induced by CCl4, as evidenced by the significant reduction in ALT, AST, ALP, and GGT levels in the LLI‐treated groups compared to the control. This protective effect is most likely attributed to the bioactive compounds found in LLI, mainly phenolics. CA, one of the major compounds detected in LLI, was specifically reported in many studies as a hepatoprotective compound against CCl_4_‐induced toxicity in murine models [26, 27]. This phenolic acid acts through several mechanisms, including interrupting the lipid peroxidation chain and increasing antioxidant enzyme activities [28]. CA also showed an antifibrosis effect by regulating the TGF‐β1/smad3 pathway in mice [27].

On the other hand, the importance of endogenous antioxidants cannot be overstated, as they play a pivotal role in the body’s defense against oxidative stress. Enzymes such as SOD and CAT are essential in detoxifying reactive oxygen species (ROS) produced during normal cellular metabolism. While natural antioxidants from plant‐based sources are often valued for their direct antioxidant activity, their benefits extend beyond this role [5]. In the present study, LLI treatment not only exerted direct antioxidant effects but also enhanced the activity of endogenous enzymes, particularly SOD and CAT, thereby strengthening the body’s antioxidant defense system. SOD converts superoxide radicals (O_2_•−) into hydrogen peroxide (H_2_O_2_), which is subsequently decomposed by CAT into water (H_2_O) and molecular oxygen (O_2_) [4]. This coordinated enzymatic action is vital for maintaining cellular homeostasis and preventing oxidative damage.

Cisplatin treatment impairs mitochondrial function and causes ROS production and lipid peroxidation, which may lead to the inactivation of antioxidant enzymes [29]. Similarly, hepatic metabolism of CCl_4_ generates highly reactive trichloromethyl (•CCl_3_) and trichloromethyl peroxy (•CCl_3_O_2_) radicals [30], leading to oxidative stress that overwhelms hepatic antioxidant defenses and significantly reduces SOD and CAT activities [31]. Regarding the lipid peroxidation assay, a significant decrease in MDA levels was shown in groups treated with LLI1 and LLI2 doses with a normal level detected in the liver with both doses, although the CCl_4_ administration. As for the kidneys, the administration of LLI2 significantly (*p* < 0.05) reduced MDA levels by nearly 60% compared to the cisplatin group. These results are in line with several studies demonstrating the positive effects of polyphenols on oxidative enzymes and on lipid peroxidation [32, 33]. To substantiate the protective role of LLI, histological evidence provided important insights into its effectiveness against toxin‐induced organ damage. The preservation of hepatic and renal architecture in LLI‐treated groups aligns with the serum biomarkers, enzymatic activity, and oxidative stress results, further supporting its antioxidant and anti‐inflammatory properties in mitigating structural damage. While this assessment demonstrated the reduction of acute tissue injury, future investigations employing specific stains such as Masson’s Trichrome or PAS could provide additional insights into potential connective tissue remodeling and fibrosis.

Recently, molecular docking has emerged as a powerful tool for understanding the nature of interactions at the molecular level, particularly in toxicology studies, where it aids in elucidating the mechanisms of compound binding and predicting potential toxic effects [34]. Results indicated that Val 146 engages in key interactions, playing a central role in stabilizing the ligand. PCA forms a strong hydrophobic interaction with the amino acid residues, while for CA, the hydrophobic interactions are predominantly focused around the residues valine (VAL 7 and VAL 146), which play a central role in stabilizing the ligand. As for naringenin, LYS 9 and ASN 51 are key contacts known for their roles in stabilizing the binding of the ligand through hydrophobic contacts, which may also facilitate further interactions, such as hydrogen bonding or ionic interactions, in conjunction with the functional groups present in naringenin. The findings are in line with the results of a previous in silico study exploring the molecular interaction between SOD and other phytochemicals, where VAL 7 and VAL146 acted as key residues in interactions between the enzyme and the ligands [35]. Similar to the ligands in this study, many phytochemicals act as allosteric activators that enhance enzyme activity [35, 36]. CA, naringenin, and PCA showed binding interactions with distinct residues within the SOD active site, a spatial distribution that may promote synergistic effects. This is supported by the observed in vivo increase in SOD activity, suggesting these phenolics work together to boost antioxidant defenses. The synergistic effects of phenolic compounds are well‐documented in various studies, particularly by the enhanced antioxidant activity observed between naringenin and CA and between PCA and CA as well [37, 38].

## 5. Conclusion

This study demonstrated the safety and antioxidant potential of *L. cylindrica* infusion in reducing oxidative damage caused by cisplatin and CCl_4_ in rats. Notably, the infusion exhibited a clear dose‐dependent protective effect, with the 40 mg/kg dose showing optimal efficacy across hepatic and renal biochemical biomarkers and histopathological architecture. This concentration‐response relationship reinforces the mechanistic reliability of the intervention and highlights its translational relevance for oxidative stress‐related conditions, including drug‐induced organ toxicity and environmental xenobiotic exposure. The absence of toxicity at therapeutically effective doses further supports the clinical applicability of this phytotherapeutic approach. While the plant’s availability and ease of preparation enhance its sustainability, the primary contribution of this work lies in providing pharmacotoxicological proof of concept for *L. cylindrica* infusions as a viable candidate for managing oxidative stress pathologies. Future investigations should focus on elucidating the specific molecular pathways involved and employing advanced histological staining to fully characterize the antifibrotic potential of this plant.

## Funding

No funding was received for this manuscript.

## Conflicts of Interest

The authors declare no conflicts of interest.

## Data Availability

The data that support the findings of this study are available from the corresponding author upon reasonable request.
